# A Patient With a Chronic Cough: An Unexpected Case of Calcium Pill Aspiration

**DOI:** 10.1177/2324709619828771

**Published:** 2019-02-06

**Authors:** Pooja Poudel, Andrew Chu, Kanish Mirchia, Manju Paul

**Affiliations:** 1 State University of New York Upstate Medical University, Syracuse, NY, USA

**Keywords:** chronic cough, calcium pill aspiration

## Abstract

Foreign body aspiration is a life-threatening medical condition that requires prompt action. Delayed diagnosis is associated with long-term serious complication often leading to death. In adults, it can remain undetected for a long period of time. The patient gives a long history of a cough, which clinicians often ignore. A chest radiograph is unreliable to exclude the disease as it may not show radiolucent objects. Diagnostic bronchoscopy is necessary to exclude the disease. We report a case of 70-year-old woman who had a 1-month history of a cough and was admitted for shortness of breath, and on further evaluation, we incidentally detected calcium tablets in her bronchus. The present case demonstrates the need for early bronchoscopy especially when the cause of a chronic cough is not known.

## Introduction

Foreign body aspiration is often overlooked as the cause of hypoxia, cough, and shortness of breath.^1^ It is rarely considered in adults with subacute or chronic respiratory symptoms unless a history of aspiration is obtained. Aspiration of pills such as iron tablets and potassium chloride can cause severe airway inflammation due to their chemical composition.^2,3^ Aspiration of calcium tablets are rarely known to cause airway obstruction, and they are rarely reported. In this article, we report a case of the incidentally detected foreign body, a calcium tablet in a patient presenting with a history of a persistent cough and shortness of breath.

## Case

A previously healthy 70-year-old woman came with a history of progressive shortness of breath and intermittent cough for 1 month. She did see her primary care provider for a cough but was evaluated further. She denied recent fever, runny nose, nasal congestion, nausea, vomiting, headache, blurry vision, chest pain, abdominal pain, urinary frequency, urgency, or lower extremity swelling.

Her vital signs on admission were the following: pulse 137 beats/min, respiratory rate 25 breaths/min, blood pressure 109/67 mm Hg, temperature 34.4°F, oxygen saturation on pulse oximeter was 70% on room air, which improved to 80% on the non-rebreather facemask. She had decreased breath sounds on the entire left lung field. She was given albuterol and duoneb nebulization and solumedrol injection. Her arterial blood gas on non-rebreather mask showed pH of 7.14, pCO_2_ of 61 mm Hg, pO_2_ of 106 mm Hg, and bicarbonate of 22 mmol/L. She was switched to noninvasive bilevel positive airway pressure ventilation for refractory hypoxia. Laboratory results showed elevated leukocytosis with white blood cell count of 22.8 × 10^3^/uL, hemoglobin of 15.3 gm/dL, and a normal blood chemistry. Respiratory panel was negative. ProBNP was elevated, 1995 pg/mL, but echocardiography showed ejection fraction of 60%, no wall motion abnormalities, and normal diastolic function. Chest X-ray showed streaky opacities at the right lung base with a small right-sided pleural effusion. Computed tomography (CT) of thorax done outside was unremarkable with no evidence of pulmonary embolism or infiltrate. She was given empiric antibiotics for suspected sepsis. Sputum culture showed only the growth of indigenous organisms.

**Figure 1.**
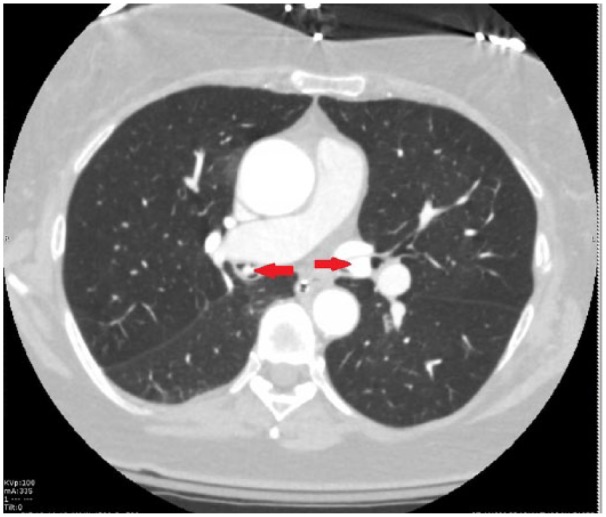
Axial view of computed tomography scan of thorax showing calcified foreign objects (red arrows) in the left and right main bronchus.

**Figure 2.**
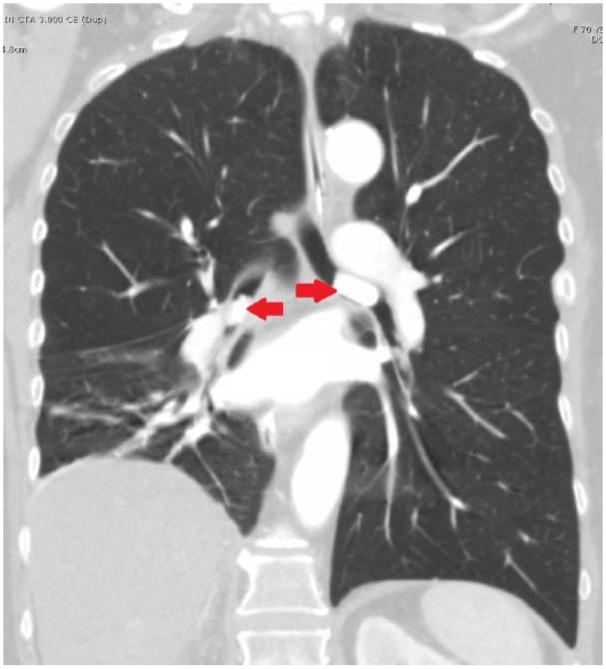
Coronal view of computed tomography scan of thorax showing calcified foreign objects (red arrows) in the left and right main bronchus.

**Figure 3.**
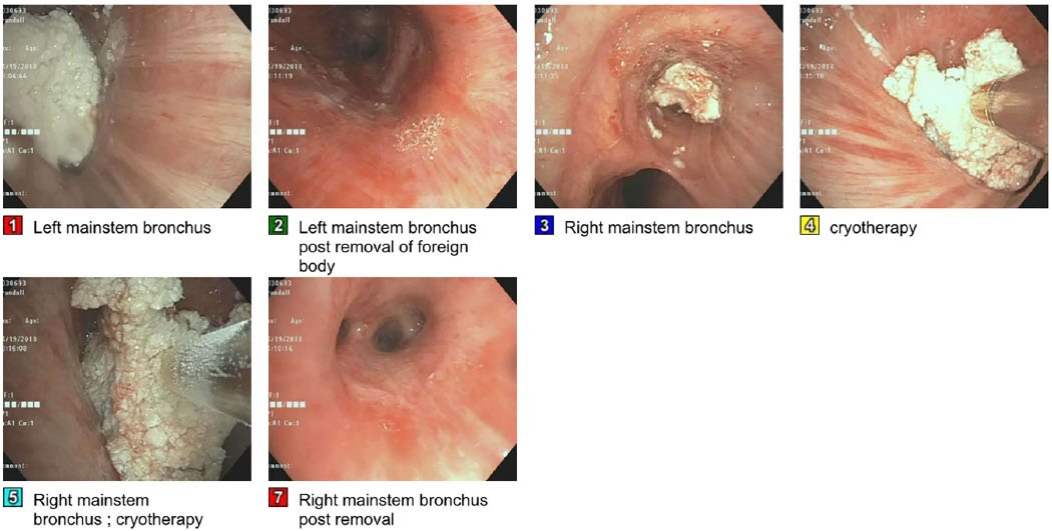
Bronchoscopy images.

The patient’s respiratory status continued to deteriorate and required intubation. A repeat CT of the thorax showed hyperdense filling defects obstructing the left main bronchus measuring 2.2 cm and in the bronchus intermedius measuring 1.5 cm (Figures 1 and 2). The patient underwent flexible bronchoscopy, which showed fragmented pill in the left mainstem bronchus and in the right bronchus intermedius, which was removed with cryotherapy (Figures 3).

**Figure 4.**
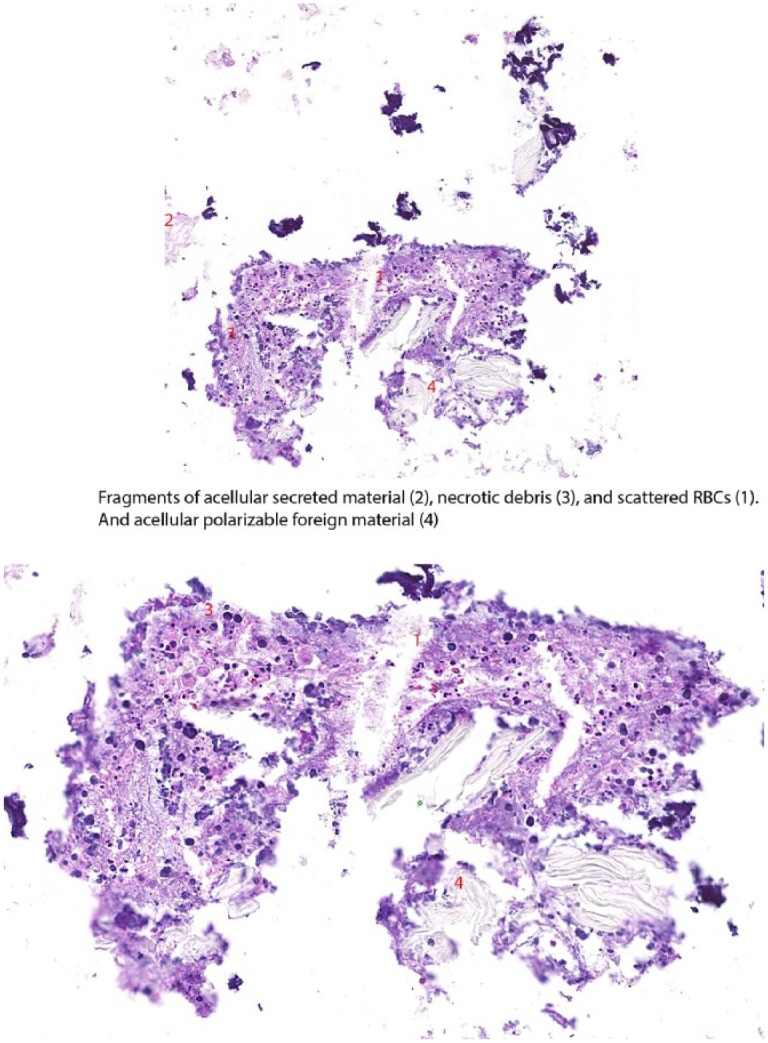
Fragments of acellular polarizable foreign material.

Pathological evaluation of the specimens revealed particles of polarizable foreign materials (Figure 4). Her respiratory status improved after the removal of the foreign body, and the patient did well with spontaneous breathing trial on ventilation and was subsequently extubated. She passed swallow evaluation and also got barium swallow studies done, which showed no aspiration. She was discharged home a few days later. Retrospectively when the patient was asked about any history of aspiration, she told that a month back she had a short duration of a cough while swallowing her calcium pill for osteoporosis.

## Discussion

Foreign body aspiration is more frequent in children than in adults. Several case series report low rate of foreign body in the airway in adults (0.66 per 100 000).^4^ In adults, it is more seen in the setting of underlying neurological disorder and alcohol and sedative use. Our patient did not have any such predisposing factors, and she did not present acutely. The presenting symptoms depend on the degree of the obstruction caused by the foreign body, location, and the length of time the foreign body has been in the airway. Chronic cough is the most common symptom followed by symptoms that mimic pneumonia including fever, chest pain, and hemoptysis. Acute or chronic cough is seen in up to 80% of all cases. Dyspnea is not common and reported by only 25% of patients in one case series of confirmed foreign body aspiration. Most patients do not recall a history of choking, which can result in the delay in diagnosis.^5^ Chest radiograph can be normal in 25% of the cases.^5^ The chest radiograph of our patient did not show any evidence of foreign body aspiration. The sensitivity of chest radiograph is 70% to 80% in children, but it has poor sensitivity in adults. Foreign bodies are mostly located in right lower lobe (28%) and about 17% in the left middle bronchus. In our case, CT scan of chest incidentally detected the presence of hyperdense filling defects in left main bronchus, which turned out to be a calcium tablet on bronchoscope evaluation. There are case reports of tablet aspiration, most common being iron pills, tetracycline, diclofenac, and ciprofloxacin. It is found that 7% of all foreign bodies aspirated in the airways are medicinal pills.^2^ Iron pill ingestion causes severe chemical injury to the tracheobronchial mucose causing mucosal necrosis and causes severe complications if not removed in time.^6^ Aspiration of calcium tablets is rarely reported, possibly due to their size.^7^ We have very few case reports of calcium pill aspiration.^8^ It is not known whether a calcium pill may spontaneously dissolve if the patient cannot cough.^2^ If the pill dissolves in the airway secretions, the diagnosis can be established by either endobronchial biopsy or bronchoalveolar lavage. What makes our case worth reporting is the delayed and innocuous presentation after aspiration of the foreign body. It emphasizes on the fact that adults may tolerate aspiration of foreign objects for a long period of time without acute life-threatening consequences.

## Conclusion

Foreign body in the airway is uncommon in adults. But if present can obstruct the airways leading to potentially serious long-term complications including recurrent pneumonia, bronchial stenosis, bronchiectasis, hemoptysis, lung abscess, pneumothorax, and pneumomediastinum. Clinicians should have this differential diagnosis in mind in patients presenting with a chronic cough because not all patients give the history of aspiration.
